# Association between functional limitation and quality of life among older adults with multimorbidity in Luxembourg

**DOI:** 10.1093/eurpub/ckac129.292

**Published:** 2022-10-25

**Authors:** P Wilk, M Ruiz-Castell, T Bohn, G Fagherazzi, K Nicholson, V Moran, TT Makovski, MN Pi Alperin, S Stranges, H Samouda

**Affiliations:** 1 Institute of Social and Preventive Medicine, University of Bern, Bern, Switzerland; 2 Department of Precision Health, Luxembourg Institute of Health, Strassen, Luxembourg; 3 Department of Epidemiology and Biostatistics, Western University, London, Canada; 4 Living Conditions, Luxembourg Institute of Socio-Economic Research, Luxembourg, Luxembourg; 5 Department of Epidemiology, Maastricht University, Maastricht, Netherlands

## Abstract

**Introduction:**

Multimorbidity, defined as the co-existence of two or more chronic conditions, is affecting an increasing number of Europeans, leading to poorer quality of life (QoL). This study assessed how functional limitation affects the QoL trajectories in a cohort of older individuals having multimorbidity, and whether there are any gender differences in these effects.

**Methods:**

We used a longitudinal cohort of 906 multimorbid respondents 50 years of age or older from Luxembourg who participated in four waves of the Survey of Health, Ageing, and Retirement in Europe (2013-2020). We used the Control, Autonomy, Self-Realization, and Pleasure scale (CASP-12) to assess QoL and the Global Activity Limitation Indicator (GALI) to measure functional limitation. Multigroup latent growth curve (LGC) modeling techniques were employed to assess how the measures of functional limitation over time are related to QoL trajectories and whether or not these effects are different by sex.

**Results:**

In 2013, over 60% of older residents of Luxembourg were affected by multimorbidity. The results from the LGC models suggest that both men and women with multimorbidity experienced a statistically significant decline in QoL between 2013 and 2020 at a constant rate; there were no significant differences in the rate of this change between men and women. The level of QoL at baseline and over time was significantly lower for individuals reporting functional limitation. However, functional limitation had no significant impact on the rate of decline in QoL for both men and women.

**Discussion and conclusions:**

As an increasing number of individuals in Europe are becoming vulnerable to more years lived with multiple chronic conditions, there is a growing need to identify factors that may lead to improvements in QoL among people affected by multimorbidity. Gaining more knowledge on the role of functional limitation may be particularly important for planning comprehensive care for patients with multimorbidity.

